# Supplementary material for: Chronobiological analysis of sex differences in electrocardiographic parameters in spontaneously breathing Wistar rats under tiletamine-zolazepam anaesthesia
Source: PLoS One. 2025 Dec 9;20(12):e0338048. doi: 10.1371/journal.pone.0338048 (PMC12688129; doi:10.1371/journal.pone.0338048)
Supplement: Attachment — Submitted filename: Response to Reviewers.pdf [file pone.0338048.s007.pdf]

Response to Reviewers

We sincerely thank the academic editor and reviewers for their thoughtful comments and constructive feedback on our manuscript titled „Chronobiological analysis of sex differences in electrocardiographic parameters in spontaneously breathing Wistar rats under Zoletil anaesthesia“. We have carefully considered all points raised and provide detailed responses below, indicating the revisions made in the manuscript where applicable.

Response to Academic editor

COMMENT: -
RESPONSE: We appreciate the time and effort taken to review our work. We would also like to inform the Academic Editor that the first author, Viktória Kapsdorferová, has changed her surname to Viktória Novotná following her marriage. This change has been reflected in the revised manuscript.

Response to Reviewer #1

COMMENT: Kapsdorferová et al. analyzed ECG parameters in Wostar rats dueing anesthesia with ZOLETIL (tiletamine and zolazepam for injection). Specifically, they studied the effects of gender and light/dark environment on the parameters. These data may be important. However, the puopose of this study is unclear and the study was not well designed.
RESPONSE: We appreciate the reviewer's critical comment. The rationale and objectives of our study are clearly explained in the Introduction and Abstract. We examined whether ECG parameters differ between the light and dark periods under Zoletil anaesthesia and between females and males under Zoletil anaesthesia. This anaesthetic combination (tiletamine–zolazepam, Zoletil) is still rarely used in rodent studies. Its cardiovascular effects are less well characterised compared to other anaesthetics, even though rats are widely employed in cardiovascular research. Therefore, we believe that our data may contribute to a better understanding of sex- and LD-dependent variability in rat cardiovascular physiology under Zoletil anaesthesia and can be helpful for future experimental studies. It should be emphasized that this study aimed to provide a numerical description of ECG parameter changes in relation to sex and LD period. We did not aim to evaluate pathological phenomena, such as atrial fibrillation, etc. This approach was considered sufficient to detect sex- and LD period-related differences.

COMMENT: #1 ZOLETIL is a brand name and it may not be suitable for the title.
RESPONSE: We agree with the reviewer that generic names are generally preferred in scientific manuscripts. Although the term *Zoletil* is commonly used in published studies (i.e., Cha et al., 2021; Chengji et al., 2022; Debbarma et al., 2024; Lu et al., 2018; Morgan et al., 2012; Svorc et al., 2016), we have carefully revised our manuscript and replaced the brand name with the generic formulation *tiletamine–zolazepam* where it was appropriate, including the title.
REFERENCES FOR THE RESPONSE:
Cha, K., Jeong, W. J., Kim, H. M., & So, B. H. (2021). Intravenous zoletil administration for the purpose of suicide. *Clinical and Experimental Emergency Medicine, 8*(2), 149.

Chengji, W. A., Qing, C. H., Hui, G. O., Jue, W. A., Yinghan, W. A., Zhengye, G. U., Xu, B. A., & Ruling, S. H. (2022). Anesthetic effects of different doses of Zoletil combined with Serazine hydrochloride on C57BL/6J mice. *Laboratory Animal and Comparative Medicine, 42*(1), 31.

Debbarma, M., Bayan, H., Konwar, B., & Sarma, K. (2024). Exploring anaesthesia options in chickens: Zoletil vs. Midazolam-Ketamine vs. Dexmedetomidine-Ketamine. *The Indian Journal of Veterinary Sciences and Biotechnology, 20*(4), 119–123.

Lu, D. Z., Feng, X. J., Hu, K., Jiang, S., Li, L., Ma, X. W., & Fan, H. G. (2018). Inductive effect of Zoletil on cystathionine β-synthase expression in the rat brain. *International Journal of Biological Macromolecules, 117*, 1211–1215.

Morgan, D. R., Scobie, S., & Arthur, D. G. (2012). Evaluation of Zoletil and other injectable anaesthetics for field sedation of brushtail possums (*Trichosurus vulpecula*). *Animal Welfare, 21*(4), 457–462.

Svorc, P., Bacova, I., Svorc Jr, P., Nováková, M., & Gresova, S. (2016). Zoletil anaesthesia in chronobiological studies. *Biological Rhythm Research, 47*(1), 103–110.

COMMENT: #2 Though the authors claimed that rat model is close to human interms of cardiac functional studies. What is the evidence?

RESPONSE: We would like to clarify that our manuscript does not state that the rat model is close to human in terms of cardiac function. On page 3, line 50, we wrote: "Rats are considered the most suitable model for studying the cardiovascular system." This statement refers to their widespread use in experimental cardiovascular research, not to direct translational applicability to humans. The term "humans" appears in our manuscript only in the following context: "P wave analysis has clinical relevance in humans; however, high-quality reference data on its morphological and temporal changes in rats are still lacking. Nevertheless, the absence of the P wave is associated with atrial fibrillation in both humans and rats " (page 14, lines 261–263). Our intention was not to generate results translatable to humans, but rather to evaluate the differences in numerical ECG parameters between light and dark periods and between male and female Wistar rats under Zoletil anaesthesia. We also acknowledge the reviewer's request for supporting evidence regarding the use of the rat model in cardiac functional studies in relation to humans. As noted by Konopelski et al. (2016), despite certain interspecies differences (e.g., the absence of a Q wave in most rat leads), there are fundamental similarities between rat and human ECG. These similarities have supported the widespread use of rat ECG in basic cardiovascular research, particularly for assessing cardiac function under physiological conditions and in experimental models of cardiovascular disease. To clarify this potential misunderstanding, we have added a short explanatory sentence with the above-mentioned reference to the Introduction section of the revised manuscript.

REFERENCES FOR THE RESPONSE:

Konopelski P, Ufnal M. Electrocardiography in rats: a comparison to human. Physiological research. 2016 Sep 1;65(5):717.

COMMENT: #3 LL68: The authors mentioned "The importance of this knowledge...." However, this mode is nothing to do with arrhythmia.

RESPONSE: We appreciate this valuable remark. As you pointed out, our publication did not address arrhythmias, nor did we claim to do so. The purpose of the theoretical introduction was primarily to emphasize the importance of understanding the effects of general anaesthesia on ECG parameters,

which subsequently serve as the basis for evaluating various pathologies, including arrhythmias (not in our case, but in general). Although assessing pathological conditions was not the aim of our study, we believe that our findings may be valuable for further experimental research on rats under Zoletil anaesthesia that explicitly focuses on this issue.

COMMENT: #4 The authors need to compare ZOLETIL with other anesthesia to measure ECG parameters. Did the author choose this med due to proarrhythmic?
RESPONSE: Our study was not designed to provide a comparative analysis of different anesthetics on ECG parameters but rather to focus specifically on Zoletil. Since Zoletil is less frequently used in rat research but more commonly applied in domestic and wild animals, its detailed effects on ECG parameters in rats remain insufficiently characterized. Nonetheless, Zoletil may represent a suitable anesthetic choice for future cardiovascular and chronobiological studies in rats due to its minimal influence on vital functions and ECG rhythmicity. Accordingly, our study analyzed the effects of sex and the light/dark cycle on ECG parameters in Wistar rats under Zoletil. We have slightly revised the Introduction to clarify the rationale for choosing Zoletil. Regarding comparisons with other anesthetics, our study focuses exclusively on Zoletil, as explained above. A broader comparative analysis of anesthetics has already been addressed in our previous review, which examined ECG parameters under various anesthetics across 123 studies (Svorc & SvorcJr., 2022).
REFERENCES FOR THE RESPONSE:
Svorc P, Svorc Jr P. General anesthesia and electrocardiographic parameters in in vivo experiments involving rats. Physiological Research. 2022 Mar 11;71(2):177.

COMMENT: #5 The QTc should be compared by other methods as well.
RESPONSE: We appreciate the reviewer’s suggestion to compare QTc using multiple correction formulas; however, the reviewer did not specify which alternative corrections should be included. Moreover, the literature on rats is not consistent regarding which QTc correction is most appropriate for this species. For example, Mulla et al. (2022) explicitly state: *"In conclusion, conflicting data still exist regarding the dependence between QT interval and heart rate in rodents. Multiple physiological and technical complexities prevent clear conclusions based on currently available data. However, a large body of evidence supports the notion that extensive use of different correction formulas may introduce significant errors, and further systematic exploration of this issue would be of high value."* In our study, we applied Bazett’s correction, which is one of the most widely used in experimental and clinical studies, including rat ECG research. Although other correction methods are available in LabChart 8, applying them to the same ECG segments can yield highly variable results (see Table 1 and Fig 1 below). Such high variability reflects differences in the underlying formulas rather than actual physiological changes and could introduce unnecessary bias. Therefore, we focused on Bazett’s correction to ensure consistency and comparability with the existing literature. Conducting a complete comparison of different QTc corrections was not the aim of our study; doing so would require the design of an entirely new study. However, we have added a note in the Discussion acknowledging the existence of alternative QTc correction formulas. We also highlighted that extensive use of different correction formulas may introduce significant errors.
REFERENCES FOR THE RESPONSE:

Mulla W, Murninkas M, Levi O, Etzion Y. Incorrectly corrected? QT interval analysis in rats and mice. Frontiers in Physiology. 2022 Oct 11;13:1002203.

Table 1: Different QTc interval corrections in Wistar rats of both sexes under tiletamine-zolazepam anesthesia during the light and dark period of the light/dark cycle.

QTc interval correction	QTc (ms)			
	**Male-light**	**Male-dark**	**Female-light**	**Female-dark**
Bazett	149,1	121,3	124,5	93,88
Framingham	187,1	172	169,1	152,3
Fridericia	126,7	101,1	108	80,59
Hodge	264,7	278,2	222,5	222
Matsunaga	98,8	77,31	85,98	63,44
Mitchell et al.	47,14	38,36	39,37	29,69
Van de Water et al.	145,6	127,8	131	111,9

ECG recordings were analyzed using LabChart 8 software (AD Instruments Ltd., CO, USA) from bipolar limb lead II at 9:00 in one male (n = 1) and one female (n = 1) during the light period of the light/dark cycle (light on: 6:00–18:00; male-light and female-light), and at 9:00 in one male (n = 1) and one female (n = 1) during the dark period of the light/dark cycle (light on: 18:00–6:00; male-dark and female-dark). For comparison of QTc intervals (ms) corrected by different methods, the same 60-second ECG segment from each individual was evaluated.

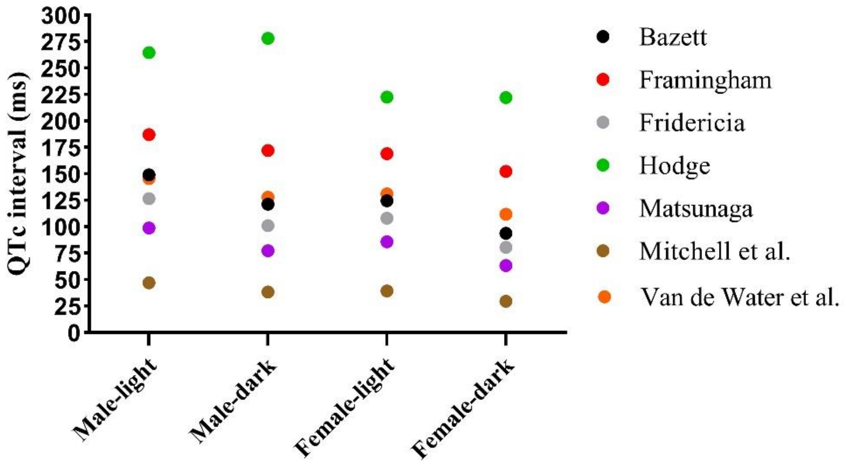

Fig. 1. Graphical presentation of different QTc interval corrections from Table 1 in Wistar rats of both sexes under tiletamine–zolazepam anesthesia during the light and dark periods of the light/dark cycle. Differences in QTc (ms) interval corrections are presented as individual data points in each experimental group (black dot – Bazett's correction; red dot – Framingham's correction; gray dot – Fridericia's correction; green dot – Hodge's correction; purple dot – Matsunaga's correction; brown dot – Mitchell et al.'s correction; orange dot – Van de Water et al.'s correction).

COMMENT: #6 It is difficult to see Table I and II. These should be combined. Data presentation is confusing in general.

RESPONSE: We appreciate the reviewer's comment regarding the presentation of Tables I and II. However, each table serves a distinct purpose: Table I summarizes the descriptive values only (mean ± SD with 95% confidence intervals) for each parameter across all experimental groups, while Table II presents the full statistical evaluation, including:

- Two-way ANOVA – p-values for the effects of sex, LD cycle period, and their interaction on all ECG parameters in all groups.
- F-test (DFn, DFd) for between-group and within-group variability for sex, LD cycle period, and their interaction.
- Sidak's post hoc tests comparing all evaluated ECG parameters between experimental groups.

Given the complexity and volume of the data, combining the tables could reduce clarity rather than enhance it. The rationale for using two-way ANOVA and Sidak's post hoc tests is clearly explained in the Methods section, allowing readers to understand the comparisons presented in Table II. For improved readability, we have slightly revised the Results section by removing detailed numerical values from the text, as these are fully reported in the tables, avoiding unnecessary repetition. Presenting the data in this manner ensures that readers can comprehensively and transparently assess the effects of sex, LD cycle, and their interaction on ECG parameters, as evaluated by two-way ANOVA, as well as any differences between the experimental groups (females–light, males–light, females–dark, males–dark) assessed using Sidak's post hoc test.

COMMENT: #7 During AF, P waves should not be observed except AF/AFL. In addition, these models did not show AF.

RESPONSE: We appreciate the reviewer's comment. As correctly noted, our model did not show AF, and this was not the purpose of our study. As mentioned above in our responses to Comments #1 and #2, our study did not aim to evaluate pathological phenomena such as AF based on morphological changes in the ECG waveform. Instead, we focused solely on the numerical description of ECG parameter changes in relation to sex and the LD period. The text in the Discussion section referring to the P wave only cites studies reporting these phenomena and was intended to provide readers with a concise explanation of the physiological and pathophysiological meaning of the analyzed ECG parameters. We have slightly revised this part of the Discussion to clarify what is derived from the literature and what represents our own results.

COMMENT: #8 In humans, it is well known that QT intervals are longer in females than in males. The authors should show actual ECG traces. Did the authors check hormonal levels?

RESPONSE: We thank the reviewer for this valuable comment. Indeed, in humans, females generally have longer QT intervals than males due to reduced repolarization reserve, which increases their susceptibility to polymorphic ventricular tachycardia of the *torsades de pointes* type, often associated with drugs prolonging ventricular repolarization (Prajapati et al., 2022). In rats, however, most *in vivo* experiments are performed exclusively in males, and therefore, valid comparative data on sex differences are often lacking, including in cardiovascular and ECG-related studies, as emphasized also in our previous work (Svorc & Svorc Jr., 2022). Regarding the present study with tiletamine–zolazepam, we did not measure hormonal levels. As described in the Methods, results were averaged irrespective of season and, in females, independently of the estrous cycle. We acknowledge this as a limitation of

our study in the Discussion section. Regarding actual ECG traces, we have now added representative examples to the Supporting Information of the revised manuscript – one for each experimental group (female–light, male–light, female–dark, male–dark). It should be noted that, due to the high interindividual variability in rats even within the same experimental group, it was challenging to select representative recordings that clearly display all significant effects. Nevertheless, we believe the inclusion of these traces increases the transparency of our results.

REFERENCES FOR THE RESPONSE:

Prajapati C, Koivumäki J, Pekkanen-Mattila M, Aalto-Setälä K. Sex differences in heart: from basics to clinics. European Journal of Medical Research. 2022 Nov 9;27(1):241.

Svorc P, Svorc Jr P. General anesthesia and electrocardiographic parameters in in vivo experiments involving rats. Physiological Research. 2022 Mar 11;71(2):177.

COMMENT: #9 I really do not understand why the authors can conclude that gender and L/D cycle are important in associations with arrhythmia. They did not show any consequences due to these differences in parameters.

RESPONSE: We thank the reviewer for this comment. We are, however, unsure where in the manuscript the reviewer found a statement suggesting that sex and the LD cycle directly affect arrhythmias in the Conclusion section, as no specific line or page was indicated. The term "arrhythmia" appears four times in the manuscript, in the following contexts:

1. **Introduction:** "Several studies also suggest an increased incidence of cardiovascular events in the early morning hours, including stroke [11,12], myocardial infarction [13–15], ventricular arrhythmias [16]…"
2. **Experimental design:** "For ECG recording, electrodes were applied subcutaneously, and the depth of anaesthesia was reassessed using the same criteria—loss of motor activity (minimal limb movement, changes in muscle tone) or cardiovascular responses to painful stimuli (alterations in HR or the onset of cardiac arrhythmias)."
3. **Discussion:** "According to the literature, the absence of the P wave is associated with atrial fibrillation in both humans and rats [25,26], and its prolongation may increase the risk of supraventricular arrhythmias in Wistar rats, particularly following myocardial infarction [27]."
4. **Discussion:** "Abnormal prolongation of the QT interval may indicate disturbances in cardiac electrical activity caused, among other factors, by toxic exposure to external agents [1], with susceptibility to drug-induced arrhythmias differing between sexes [31]."

If we have overlooked a statement that led the reviewer to this interpretation, we would be grateful for further clarification. In any case, our conclusions are based strictly on the performed statistical analyses, with results interpreted in the context of a two-way ANOVA. The effects of LD cycle, sex, and their interactions were assessed using two-way ANOVA, with Sidak's post hoc test for group comparisons. The detailed statistical results supporting these conclusions are presented in Table 2. As repeatedly stated and additionally clarified in the Discussion, we only analyzed numerical ECG parameters and did not evaluate pathological phenomena based on morphological changes in the ECG waveform.

Response to Reviewer #2

COMMENT: Manuscript nicely drafted and thorough statistical analysis done vigorously and appropriately. Manuscript has been written in standard English. Author has made all data available in the manuscript for review.
RESPONSE: We sincerely thank the reviewer for the positive evaluation of our manuscript and for acknowledging the thoroughness of our statistical analyses and the clarity of the writing. We appreciate the time and effort taken to review our work.

We thank the editor and all reviewers for their valuable feedback and constructive suggestions. We believe that the revisions and clarifications made have substantially improved the manuscript and addressed all raised concerns. We hope that the revised manuscript is now suitable for publication in PLOS ONE.

Sincerely,
Pavol Švorc, Prof. Dr. PhD
Corresponding author
Department of Medical Physiology
Faculty of Medicine, Pavol Jozef Šafárik University
Trieda SNP 1, 040 11 Košice, Slovakia
Email: pavol.svorc@upjs.sk
